# Slow mitochondrial repair of 5′-AMP renders mtDNA susceptible to damage in APTX deficient cells

**DOI:** 10.1038/srep12876

**Published:** 2015-08-10

**Authors:** Mansour Akbari, Peter Sykora, Vilhelm A. Bohr

**Affiliations:** 1 Center for Healthy Aging, SUND, University of Copenhagen, Denmark; 2 Laboratory of Molecular Gerontology, National Institute on Aging, 251 Bayview Blvd, Baltimore, USA

## Abstract

Aborted DNA ligation events in eukaryotic cells can generate 5′-adenylated (5′-AMP) DNA termini that can be removed from DNA by aprataxin (APTX). Mutations in *APTX* cause an inherited human disease syndrome characterized by early-onset progressive ataxia with ocular motor apraxia (AOA1). APTX is found in the nuclei and mitochondria of eukaryotic cells. Depletion of APTX causes mitochondrial dysfunction and renders the mitochondrial genome, but not the nuclear genome susceptible to damage. The biochemical processes that link APTX deficiency to mitochondrial dysfunction have not been well elucidated. Here, we monitored the repair of 5′-AMP DNA damage in nuclear and mitochondrial extracts from human *APTX*^+/+^ and *APTX*^−/−^ cells. The efficiency of repair of 5′-AMP DNA was much lower in mitochondrial than in nuclear protein extracts, and resulted in persistent DNA repair intermediates in APTX deficient cells. Moreover, the removal of 5′-AMP from DNA was significantly slower in the mitochondrial extracts from human cell lines and mouse tissues compared with their corresponding nuclear extracts. These results suggest that, contrary to nuclear DNA repair, mitochondrial DNA repair is not able to compensate for APTX deficiency resulting in the accumulation of mitochondrial DNA damage.

APTX belongs to the histidine triad (HIT) superfamily of nucleotide hydrolases and transferases^1^. Frame-shift, splice-site, nonsense and missense mutations identified in the *APTX* cluster primarily in the HIT domain, destabilize AOA1 protein, and cause the human hereditary neurodegenerative disease, ataxia with oculomotor apraxia (AOA1)^2^,^3^,^4^. APTX resolves 5′- AMP termini that can occur during premature termination of DNA ligation events in DNA repair and replication^3^,^5^,^6^. Unlike many DNA repair deficiency disorders, patients with AOA1 are not susceptible to cancer nor are APTX deficient cells hypersensitive to genotoxic agents^7^,^8^,^9^,^10^. APTX localizes to the nuclear and mitochondrial compartments of human cells^10^, and depletion of APTX causes mitochondrial dysfunction and susceptibility to mtDNA damage. Proper mitochondrial function is especially critical for neuronal cells, because of high energy demand^11^. Mitochondria play key roles in energy metabolism, fatty acid catabolism, calcium homeostasis, apoptosis, cell proliferation and autophagy^12^,^13^. Defects in mitochondrial homeostasis cause a number of severe and phenotypically variable diseases including ataxic neurodegenerative disorders such as Friedreichs ataxia and Neuropathy, ataxia, and retinitis pigmentosa (NARP)^13^,^14^. However, recent studies also suggest that defects in repair of the nuclear genome can contribute to mitochondrial dysfunction, leading to neurodegeneration^15^,^16^,^17^,^18^, indicating that defects in either nuclear and mitochondrial DNA repair can directly or indirectly lead to mitochondrial dysfunction. This study explores the possibility that APTX actively promotes repair of mtDNA damage. In particular, a biochemical approach was used to determine the rate of repair of 5′-AMP ssDNA breaks in human nuclear and highly purified mitochondrial extracts. The results show that APTX deficiency impairs repair of 5′-AMP damaged DNA substrates in mitochondrial extracts, suggesting that APTX plays a functional role in DNA repair in the mitochondrial compartment, and establishing a putative connection between mitochondrial dysfunction and AOA1 pathology.

## Materials and Methods

Synthetic oligonucleotides were from TAG Copenhagen. [γ-^32^P] ATP, [α-^32^P]dCTP, and [α-^32^P]dGTP were from Perkin Elmer. Restriction enzymes and the 5′ DNA adenylation kit were from New England Biolabs. Complete Protease inhibitor was from Roche.

### Mitochondrial disease database

Using the mitochondrial disease database (mitodb), symptoms of AOA1 were compared to diseases commonly regarded as mitochondrial, non- mitochondrial or unclear in origin^19^.

### Preparation of mitochondrial and nuclear extracts

C3ABR (APTX proficient) and L938 (APTX deficient) lymphoblast cell lines were grown in RPMI 1640 medium with 20% fetal calf serum and 0.1 mg/ml gentamicin in 5% CO_2_. L938 is a Epstein-Barr virus transformed AOA1 patient derived lymphoblast cell line that carry a P206L/P206L mutation in the histidine triad (HIT) domain of APTX. This mutation destabilizes the APTX protein^10^,^20^. U2OS cells were cultured in Dulbecco’s modified Eagle’s medium (DMEM-Glutamax, Gibco), with 10% fetal calf serum and 0.1 mg/ml gentamicin in 5% CO_2_. Mitochondrial extracts were prepared as described previously^21^. Cells were collected by centrifugation at 400 × *g*, suspended in hypotonic buffer (20 mM HEPES-NaOH pH 7.4, 5 mM KCl, 1 mM DTT, and Complete protease inhibitor) and incubated on ice until swollen. 2 × MSH buffer (420 mM mannitol, 140 mM sucrose, 20 mM HEPES pH 7.4, 4 mM EDTA, 2 mM EGTA, and 5 mM DTT) was then added (1:1, v/v) and the cells were broken in a tight Dounce homogenizer. The homogenate was centrifuged at 1,000 × *g*. This step was repeated until no nuclei were seen in the pellet (typically 2–3 times). The crude mitochondria were pelleted at 10,000 × *g* for 30 min, suspended in 1 × MSH/50% Percoll and loaded on top of a 1 × MSH/50% Percoll mixture and centrifuged at 50,000 × *g* for 75 min. Mitochondrial fraction was removed from the gradient and centrifuged in 1 × MSH buffer at 3,000 × *g* for 10 min to remove Percoll. The mitochondria were suspended in buffer 10 mM HEPES, pH 8.0 and 200 mM KCl, and then in an identical volume of a lysis buffer (10 mM HEPES, pH 8.0, 200 mM KCl, 2 mM EDTA, 2 mM DTT, 20% glycerol, 1% IGEPAL, 1% Triton X-100, and Complete protease inhibitor), kept on ice for 60 min and mildly sonicated three times at 5 W for 3 s, with 30 s intervals. Mitochondrial debris was removed by centrifugation at 21,100 × *g* for 15 min, and the supernatant was collected and stored at –80 °C. Mitochondria from mouse brain and liver were isolated as described before^22^,^23^, with minor modifications. Briefly, frozen whole brain/liver were thawed in 5 ml of ice-cold 1 × MSH, cut into small pieces, homogenized in a loose Dounce homogenizer and centrifuged at 1000 × *g* for 10 min. The supernatant was collected and centrifuged at 10,000 × *g* for 10 min. The supernatant was carefully removed and the pellet was suspended in 1 × MSH/50% Percoll and loaded on top of a 1 × MSH/50% Percoll mixture and centrifuged at 50,000 × *g* for 75 min. The mitochondrial fraction was removed from the gradient and mitochondrial extracts were prepared as described above. For nuclear extracts, after breaking the cells with Dounce homogenizer, the nuclei were collected at 400 × *g* for 10 min and lysed in the above lysis buffers.

### Western blot analysis (WB)

20 μg protein extracts were separated in 12% Tris-glycine SDS-PAGE (Invitrogen). The following primary antibodies were used: Aprataxin (ab31841, Abcam), PCNA (sc-56, Santa Cruz), Lamin (sc-6215, Santa Cruz), COX 4 (sc-133478, Santa Cruz), VDAC-1 (sc-58649, Santa Cruz), TFAM (B01P, Abnova). The secondary antibodies, polyclonal rabbit-mouse IgG/HRP or peroxidase-labeled polyclonal swine-rabbit IgG were from Dako Cytomation.

### DNA substrates

**Figure 1:**
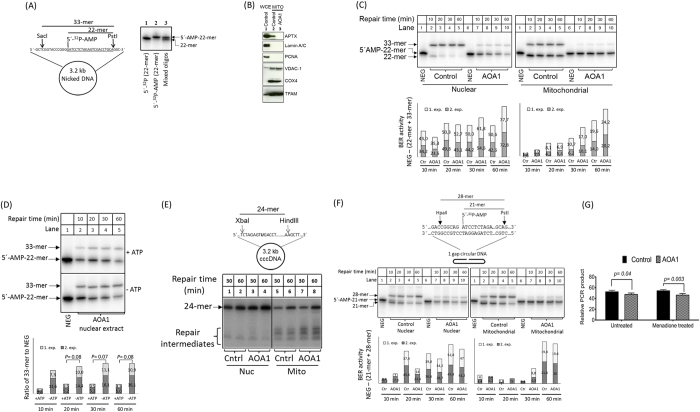
(**A**) Schematic diagram of DNA substrate for 5′-AMP repair analysis. 22-mer oligonucleotide was labeled at 5′-end with ^32^P (lane 1) followed by 5′-adenylation (lane 2). 5′-end labeled oligonucleotide was annealed to a circular single-stranded DNA and DNA synthesis was carried out to prepare double-strand circular DNA containing a nick with 5′-AMP, at a specific position. The nucleotide sequence of the 22-mer oligonucleotide is underlined. Mixed oligos from lanes 1 and 2 were separated as distinct bands in 20% denaturing polyacrylamide gel. (**B**) Western blot analysis of whole and mitochondrial extracts from C3ABR (control, lanes 1 and 2, respectively), and mitochondrial extract from L938 (AOA1, lane 3) lymphoblast cell lines. (**C**) Repair analysis of 5′-AMP DNA in nuclear extracts (left) and mitochondrial extracts (right). The upper band (33-mer) corresponds to the ligated products. Combined 33-mer signal and removal and loss of 5′-^32^P signal during repair DNA synthesis (22-mer) relative to control (lanes 1 and 6) was used to measure the rate of SSBR of 5′-AMP DNA (diagram). The experiments were conducted in duplicate and the result of each experiment is shown in the stacked column (**D**) Direct ligation of 5′-AMP by non-adenylated DNA ligase. Repair analysis was performed in nuclear extracts from APTX deficient AOA1 cells in the presence or absence of additional ATP in the reaction as indicated. The diagram shows the level of directly ligated nick 5′-AMP from two independent experiments. An increase in the level of 5′-AMP ligation in the reaction (33-mer) without additional ATP demonstrates that non-adenylated DNA ligase molecules in the extracts likely carry out the direct ligation of nick 5′-AMP DNA. The experiments were carried out in duplicate as indicated (exp. 1 and 2). Statistical significance of apparent increased level of the 33-mer bands was determined by T-test using Excel. (**E**) Uracil-BER analysis of the nuclear and mitochondrial extracts. The upper band (24-mer) corresponds to fully repaired DNA. Repair intermediates are indicated. (**F**) Analysis of nuclear and mitochondrial 5′-AMP repair using 1-gap substrate. DNA repair reactions were carried out at 30 °C in 20 μg extracts, at the indicated times. The repaired DNA was purified from extract, digested with HpaII and PstI and separated in denaturing polyacrylamide gel. The graphs show the results of two independent experiments as in C. (**G**) MtDNA damage analysis. The relative amounts of mtDNA damage in L938 (APTX deficient, AOA1) to C3ABR (APTX proficient, control) cells without or with menadione treatment was assessed by PCR amplification of an 8.9 kb fragment of mtDNA. PCR was carried out in triplicate. Error bars are SEM. T-tests were performed to determine the statistical significance of the apparent difference in the level of mtDNA damage in control and AOA1 cells. Statistical analyses were performed using Excel.

Double-stranded circular DNA substrate containing a nick with 5′-AMP at a specific position was prepared as follows: oligonucleotide 5′-GATCCTCTAGAGTCGACCTGCA-3′ was radio-labeled at the 5′ end by [γ-^32^P]ATP and T4 polynucleotide kinase, and adenylated to generate an oligonucleotide with 5′-AMP. The adenylated oligo was annealed to single-strand DNA derived from pGEM-3Zf(+) plasmid (Fig. 1A) and DNA synthesis was carried out with T4 gene 32 single-strand DNA binding protein, T4 DNA polymerase, and dNTPs at 37 °C for 120 min^24^. For cold DNA substrate, the same oligonucleotide was 5′-phosphorylated with ATP, adenylated and DNA synthesis was carried out as above. Double-stranded closed-circular DNA containing uracil at a specific position was prepared as described previously^22^,^24^.

For DNA substrate containing one nucleotide gap adjacent to 5′-AMP DNA, a 68-mer oligo (5′-P-GACGGCCAGTGTCACTGGCCGTCCTAGGAGATCTCAGCTGGACGTCTGCCGGTCACTGTGACCGGCAG-3′) was annealed to the 5′- ^32^P-AMP 21-mer oligo (5′-ATCCTCTAGAGTCGACCTGCA-3′). The substrate is designated 1 gap circular DNA.

### DNA repair assays

Repair reaction was carried out in 20 μg extract in final 40 mM HEPES-KOH pH 7.8, 1 mM DTT, 5 mM MgCl_2_, 120 mM KCl, 2 mM ATP, 0.36 mg/ml BSA, 20 μM of each dNTPs, 4.5 mM phosphocreatine, 50 ng/μl creatine kinase, 8 nM DNA substrate, at 30 °C for the indicated times in a volume of 25 μl. The reaction was stopped by adding EDTA and further incubated with SDS and proteinase K at 42 °C for 30 min. DNA was purified with phenol/chloroform extraction and salt precipitation, suspended in 10 mM Tris-HCl pH 8.5 and digested with the indicated restriction enzymes and separated in 20% denaturing polyacrylamide gel at 400 V for 2 h. The repair experiments were carried out in duplicates in two independently prepared nuclear and mitochondrial extracts.

SSBR analysis was carried out as above, but in 20 μM dATP, 20 μM dTTP, 20 μM dCTP, 5 μM dGTP, and 80 nCi/μl dGTP at 30 °C for the indicated times in a volume of 25 μl. For uracil-BER, 5 μM dCTP and 80 nCi/μl [α-^32^P]dCTP were used in the reaction. DNA was purified from the extracts, digested with the indicated restriction enzymes and separated in denaturing polyacrylamide gel.

### DNA damage analysis

Total cellular DNA was extracted using the QIAamp DNA mini kit (QIAGEN). DNA was quantified using Epoch Microplate Spectrophotometer (Bio-Tek). PCR primers were previously described^25^. For mtDNA damage analysis we used forward primer; 5′-TCTAAGCCTCCTTATTCGAGCCGA-3′, and reverse primer; 5′-TTTCATCATGCGGAGATGTTGGATGG-3′, to amplify an 8.9 kb region of mtDNA. The basic idea for this experiment is that the presence of damage in a DNA template halts polymerase elongation resulting in a lower amount of PCR product compared with an undamaged DNA template^25^. The PCR reaction contained; 10 ng DNA template, 20 pmol of each primer, 0.25 mM of each dNTPs, and 1 unit of TaKaRa EX Taq polymerase, in 50 μl reaction. PCR was carried out for 20 cycles; 94 °C 1 min, 94 °C 15 sec, 63 °C 30 sec, 65 °C 9 min (15 min for β-globin), 72 °C 10 min, in triplicate. Notably, at 20 cycles PCR was within the exponential phase (Fig. S3A). 40 μl of each PCR product was separated in four lanes (10 μl in each lane) in 1% agarose gel and the relative amplification was determined by measuring the intensity of the bands using Image J software. For nuclear DNA damage analysis, the forward primer; 5′-CGAGTAAGAGACCATTGTGGCAG-3′, and reverse prime; 5′-GCACTGGCTTAGGAGTTGGACT-3′, were used to PCR amplify a 13.5 kb region of the β-globin gene. To ensure that the amplification of the long-PCR was not affected by possible differences in mtDNA steady state levels, a 179 bp fragment of mtDNA was amplified, because the amplification of this small fragment should not be affected by damage. The primers used were, forward; 5′-GCAGCCCTAGCAACACTCC-3, and reverse primer; 5′-GAGGTCTGGTGAGAATAGTGT-3′. PCR was carried out in 10 ng DNA template, 10 pmol of each primer, 0.2 mM of each dNTPs, and 1 unit TaKara DNA polymerase in a final volume of 50 μl. PCR was run for 20 cycles at; 94 °C 1 min, 94 °C 30 sec, 57 °C 30 sec, 72 °C 30 sec, 72 °C 5 min. The PCR products were separated in 2% agarose gel and the bands were quantified as above. This approach turned out to provide a semi-quantitative, rapid and reliable method to assess DNA damage.

## Results

Aprataxin has both mitochondrial and nuclear localization and mutations in the protein lead to the neurodegerative disorder AOA1^10^. We used a previously described disease database to determine whether AOA1 had similarities to other mitochondrial disorders^19^. Based on symptoms of the disease including ataxia, cognitive decline, dysarthria, neuropathy, oculomotor apraxia, chorea, and muscle weakness^26^, AOA1 clearly clustered with diseases known to have a mitochondrial origin (Fig. S1). AOA1 clustered most closely with neurodegenerative diseases Friedreichs ataxia and Neuropathy, ataxia, and retinitis pigmentosa (NARP) (Fig. S1), which have clear mitochondrial disease origins. Despite the role of aprataxin in nuclear DNA repair, symptoms of AOA1 did not cluster with known diseases caused by the breakdown of nuclear DNA repair. We asked whether the observation that AOA1 presents with primarily mitochondrial symptoms correlated with the protein being more important in this organelle.

The repair of 5′-AMP in nuclei and mitochondria of human cells was measured by incubating a dsDNA circular DNA substrate with a single site-specific 5′-^32^P-AMP-modified nick (Diagram as Fig. 1A) in the presence of highly-purified mitochondrial or nuclear extracts from control (*APTX*^+/+^) or AOA1 (*APTX*^-/-^) patient derived human lymphoblast cells. Reaction products were cleaved with restriction enzymes and analyzed by denaturing polyacrylamide electrophoresis (PAGE), under conditions that resolve unrepaired 5′-AMP from repaired 5′-phosphorylated (5′-P) DNA (Fig. 1A, gel). The purity of cell derived mitochondrial extracts was examined by Western blot analysis (Fig. 1B). Nuclear proteins, Lamin A/C and PCNA were not detected in the mitochondrial extracts indicating that the mitochondrial extracts were free of nuclear proteins.

In the APTX proficient control nuclear extract, the 5′-AMP-22-mer DNA substrate was efficiently processed, such that no 5′-AMP residue was detected after 10 min (Fig. 1C left, lane 2). As expected, 5′-AMP was not removed from DNA in the AOA1 extract (Fig. 1C left, lanes 7–10, 5′-AMP-22-mer). However, ~5% of the substrate was directly ligated (Fig. 1C left, lanes 7–10, 33-mer, and Fig. S2A, AOA1). The likely explanation for this observation is that the 5′-AMP DNA substrate is recognized and processed by non-adenylated DNA ligase as a normal reaction intermediate, so that the reaction cycle proceeds to completion. This hypothesis was confirmed by the observation that a higher amount of 33-mer product was generated in reactions without exogenous ATP (Fig. 1D). Figure 1C shows that the level of direct ligation of 5′-AMP-repaired DNA did not change following prolonged incubation (33-mer), while the intensity of ^32^P-labelled 22-mer was progressively reduced by longer incubation time. This likely reflects that the repair of 5′-AMP in nuclear extracts takes place via several mechanisms; 1) ligation of 5′-AMP-DNA by non-adenylated DNA ligase molecules, 2) a rapid direct ligation of the nick following the removal of 5′-AMP from DNA (in APTX proficient cells), and, 3) slower but progressive alternative repair pathways such as SSBR or long-patch BER (LP-BER) are active on 5′-AMP-DNA termini in both APTX-proficient and APTX-deficient nuclear extracts.

The repair of 5′-AMP nicked DNA was also assessed in mitochondrial extracts, shown as Fig. 1C right. In mitochondrial extract from control cells, approximately 25% of the nicks were ligated after the removal of 5′-AMP (Fig. 1C right, lanes 2–5, 33-mer, and Fig. S2B, control), and a comparable amount of the nicks were left unligated (Fig. 1C right, lanes 2–5, 22-mer). Similar to AOA1 derived nuclear extracts, the 5′-AMP was not removed from DNA in the AOA1 mitochondrial extracts, and a small fraction of 5′-AMP was directly ligated (Fig. 1C right, lanes 7–10, 33-mer, and Fig. S2B, AOA1). However, mitochondrial SSBR was significantly less efficient, contributing to the repair of only ~25% of the substrate after 60 min repair time (compare the diagram in Fig. 1C right to the diagram in Fig. 1C left). This was unexpected because previous studies showed relatively robust LP-BER/SSBR activity in mitochondrial extracts from human cells^21^,^27^,^28^, which was confirmed by measuring repair of uracil in circular dsDNA in the presence of mitochondrial extracts from control and AOA1 cells (Fig. 1E, 24-mer). The repair of uracil-DNA was more efficient in nuclear than in mitochondrial extracts, and repair intermediates were more abundant in mitochondrial extracts. These repair intermediates typically arise as a result of DNA ligation failure^21^,^22^, which is the rate-limiting step in mitochondrial BER^22^. The results confirm that long-patch repair is functional in the mitochondrial extracts and that the low rate of 5′-AMP repair (Fig. 1C right) was not because of a defect in long-patch repair activity in the extracts.

To rule out that nicked DNA may somehow impede the repair of 5′-AMP by nuclear and mitochondrial BER, we next used a substrate containing a 1 nucleotide gap adjacent to 5′-AMP. Comparable results were obtained when the DNA substrate carried a 1 nucleotide gap instead of a nick (compare Fig. 1F with Fig. 1C). These results indicate that mitochondrial and nuclear DNA repair can access 5′-adenylated DNA in both a nick and when it is situated adjacent to a 1 nucleotide gap.

We used a PCR based method to assess whether the reduced mitochondrial repair capacity of AOA1 cells would lead to an accumulation of mtDNA damage. Cells were treated with 25 μM menadione for an hour and allowed to recover for 12 hours. Total DNA was then isolated from treated and untreated cells and a 8.9 kb region of mtDNA was amplified by PCR with the assumption that the efficiency of PCR synthesis would correlate with the amount of DNA damage in the template DNA^25^. The results suggest that endogenous and menadione-induced mtDNA lesions are more abundant in AOA1 than in control cells (Fig. 1G). It is unlikely that this reflects depletion of mtDNA in L938 cells, based on a control experiment using a 179 bp PCR amplicon (Fig. S3C). In contrast, the abundance of lesions in nuclear DNA appeared to be similar in the two cell lines (Fig. S3D).

**Figure 2:**
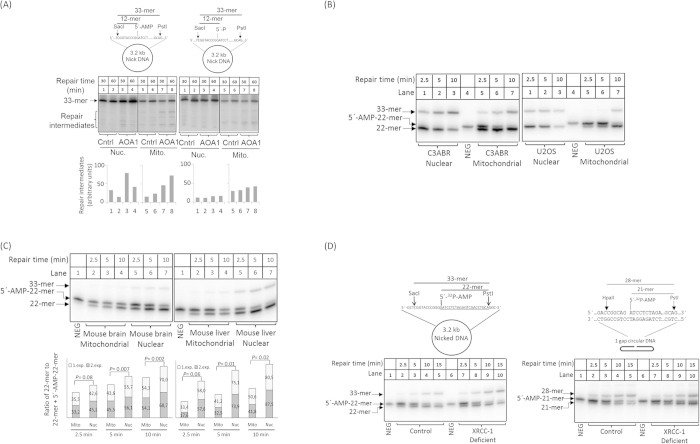
(**A**) Analysis of SSBR of nick 5′-AMP. Repair reactions were carried out in the presence of [α-^32^P]dGTP. A substrate containing a normal nick was used as control (right panel). Schematic presentations of DNA substrates are included. The diagrams show the relative level of repair intermediates. (**B**) Time-course analysis of 5′-AMP removal activity. Mitochondrial and nuclear 5′-AMP removal activity of human C3ABR and U2OS cell lines was measured using DNA substrate shown in Fig. 1A. The repair reaction was carried out in 20 μg extract at 30 °C for the indicated times. (**C**) Time-course analysis of 5′-AMP removal activity in mitochondrial and nuclear extracts from mouse brain and liver using DNA substrate shown in Fig. 1A.The experiments were carried out in duplicate and the result of each experiment is shown in stacked column (exp. 1 and 2). Statistical significance of the changes was determined by T-test using Excel. (**D**) A possible effect of Xrcc1 in APTX activity was analyzed in nuclear extracts from Xrcc1 proficient AA8 (control, lanes 2–5) and Xrcc1 deficient EM9 (lanes 7–10) CHO cell lines using the 3.2 kb (left image) and 1 gap circular (right image), DNA substrates, respectively.

Despite a significant difference in DNA damage levels in the human lymphocytes, the accumulation of DNA damage was lower than reported in other APTX deficient cell types^10^, suggesting more robust axillary repair mechanisms in the absence of AOA1. To further investigate the role of alternate repair pathways in repair of 5′-AMP-DNA termini, unlabeled nicked 5′-AMP circular dsDNA was incubated in nuclear or mitochondrial extracts in the presence of ^32^P-radiolabeled dNTPs. Repair of the repair intermediates was considerably slower in the presence of nuclear extracts from AOA1 cells than from control cells (Fig. 2A, compare repair intermediates in lane 1 with lane 3), with some repair intermediates detected after 60 min (compare lanes 3 and 4). Repair efficiency was also lower in AOA1 mitochondrial extracts, with more abundant repair intermediates detected after 60 min (Fig. 2A, left, compare lanes 5 and 6, with lanes 7 and 8, respectively). Nicked 5′-P DNA was used as control (Fig. 2A, right) and was efficiently repaired in both control and AOA1 nuclear extracts (compare lanes 1–4 in Fig. 2A, right, to lanes 1–4 in Fig. 2A, left). These results suggest that in the absence of APTX, 5′-AMP-DNA termini are not efficiently repaired by alternate repair pathways in the mitochondrial compartment.

The kinetics of repair of nuclear and mitochondrial 5′-AMP was studied in mitochondrial and nuclear extracts from human lymphocyte (C3ABR) and osteosarcoma (U2OS) cell lines, and mouse brain and liver. The results show that 5′-AMP removal was very efficient (100% repair) in nuclear extracts from C3ABR and U2OS cells (Fig. 2B). In contrast, in mitochondrial extracts, the removal of 5′-AMP was significantly slower. Likewise, in mouse brain and liver mitochondrial extracts, the removal of 5′-AMP was slower than in corresponding nuclear extracts (Fig. 2C).

X-ray repair cross-complementing protein 1 (XRCC1) is a non-enzymatic nuclear protein that facilitates BER/SSBR by interacting with multiple DNA repair proteins. APTX interacts with XRCC1 and has been identified in a XRCC1-containing protein complex^8^,^9^,^29^. However, it is not known whether this interaction influences the activity of APTX. To test whether XRCC1 stimulates APTX-mediated repair of 5′-AMP termini and whether the absence of XRCC1 in mitochondria^30^ may account for the slow 5′-AMP repair in mitochondria, we conducted 5′-AMP repair analysis in nuclear extracts from EM9 Chinese hamster ovary cells (Xrcc1-deficient) and the corresponding Xrcc1-proficient cell line AA8. We found no differences in repair efficiency between the extracts (Fig. 2D). This suggests that XRCC1 does not modulate the efficiency of repair of 5′-AMP, and the observed slow mitochondrial removal of 5′-AMP is not directly related to the absence of XRCC1 in mitochondria.

## Discussion

In this study we have undertaken a biochemical approach to investigate the strong mitochondrial phenotype associated with the hereditary neurodegenerative disorder AOA1. We examined the efficiency of repair of 5′-AMP DNA termini in extracts from wild type and mutant *APTX* cells and demonstrated that 5′-AMP DNA termini are repaired less efficiently in mitochondrial than in nuclear extracts.

Biochemical analysis of 5′-AMP DNA repair was previously investigated in AOA1 whole cell extracts^31^,^32^. Although our results support the general conclusions presented in those reports, some important differences were noted. 1) Under the experimental conditions used in the present study, approximately 75% of 5′-AMP termini were repaired by SSBR in APTX-deficient nuclear extracts (Fig. 1C,F), while this role of SSBR was not reported in previous studies. This discrepancy might reflect the use of a circular dsDNA substrate in the present study, potentially because circular dsDNA is relatively more resistant to exonucleolytic degradation than linear DNA substrates. Alternatively, nicked circular dsDNA might recruit SSBR proteins more effectively than other DNA substrates^33^. 2) This study provides evidence that non-adenylated DNA ligase facilitates ligation of 5′-AMP DNA termini. This observation was not reported in previous studies, although a similar idea was explored by adding recombinant T4 DNA ligase to 5′-AMP repair assays. The results of the present study suggest that non-adenylated DNA ligase could potentially promote repair of 5′-AMP DNA termini *in vivo*. This may be particularly important in post-mitotic cells, where SSBR activity is low^34^,^35^. 3) Here, we analyzed nuclear and mitochondrial repair of 5′-AMP and found significant differences in the capacity for repair of 5′-AMP termini in the nuclear and mitochondrial compartments. 4) APTX-deficient nuclear extracts process intermediates of 5′-AMP repair slowly, while such intermediates appear to persist for a longer time in mitochondrial extracts of the same cell type (Fig. 2A).

Ribonucleotides are frequently mis-incorporated into genomic DNA^36^. RNase H2 cleaves the DNA on the 5′side of the RNA-DNA junction generating an estimated 1,000,000 nicked RNA-DNA junctions per cell cycle^37^. Attempts to ligate such nicks can generate 5′-AMP-RNA-DNA lesions. APTX deadenylates these junctions, and this lesion evoked S-phase checkpoint activation and a growth defect in APTX-deficient yeast^38^. DNA polymerase γ can incorporate ribonucleotides into DNA^39^,^40^, and ribonucleotides have been detected in mtDNA^41^. Of the two RNases H1 and H2 in human cells, only RNase H1 localizes to mitochondria^42^. RNase H1 and RNase H2 have distinct cleavage specificities. Only RNase H2 can repair single nucleotides in DNA, but both RNase H1 and H2 can cleave long stretches of RNA/DNA hybrids^42^. Additional studies are needed to determine what mechanism recognizes and removes singlet ribonucletides in mtDNA.

Further investigation into the consequences of persistent mis-incorporated ribonucleotides into mtDNA and the rate of formation of 5′-AMP-RNA-DNA lesions that can disrupt mtDNA replication and transcription and contribute to mitochondrial dysfunction in AOA1 patients is warranted.

Recently, the nuclear DNA polymerase β (Polβ) was shown to efficiently remove 5′-AMP-dRP from DNA^43^. The level of Polβ may increase in non-diving cells^44^. Moreover, Polβ in cooperation with FEN1, can carry out LP-BER in non-dividing cells^45^,^46^, and as such may contribute to the repair of 5′-AMP in post-mitotic cells. Thus, consistent with our results, in the nucleus, several processes could promote repair of 5′-AMP DNA damage^6^,^47^. These results may explain the absence of cancer predisposition in AOA1 patients, the lack of overt phenotype in the APTX null mice, and the relative resistance of APTX-deficient cells to DNA damaging agents.

Based on the findings of this study, additional analysis of the role of mitochondrial dysfunction in AOA1 pathology is warranted. A fresh focus on the mitochondrial aspects of the debilitating disorder may provide patients with AOA1 with a therapeutic avenue.

## Additional Information

**How to cite this article**: Akbari, M. *et al.* Slow mitochondrial repair of 5′-AMP renders mtDNA susceptible to damage in APTX deficient cells. *Sci. Rep.* **5**, 12876; doi: 10.1038/srep12876 (2015).

## Supplementary Material

Supplementary data

## References

[b1] Kijas A. W., Harris J. L., Harris J. M. & Lavin M. F. Aprataxin forms a discrete branch in the HIT (histidine triad) superfamily of proteins with both DNA/RNA binding and nucleotide hydrolase activities. J Biol Chem 281, 13939–13948 (2006). 16547001 10.1074/jbc.M507946200

[b2] Caldecott K. W. Single-strand break repair and genetic disease. Nat Rev Genet 9, 619–631 (2008). 18626472 10.1038/nrg2380

[b3] Date H. *et al.* Early-onset ataxia with ocular motor apraxia and hypoalbuminemia is caused by mutations in a new HIT superfamily gene. Nat Genet 29, 184–188 (2001). 11586299 10.1038/ng1001-184

[b4] Moreira M. C. *et al.* The gene mutated in ataxia-ocular apraxia 1 encodes the new HIT/Zn-finger protein aprataxin. Nat Genet 29, 189–193 (2001). 11586300 10.1038/ng1001-189

[b5] Ahel I. *et al.* The neurodegenerative disease protein aprataxin resolves abortive DNA ligation intermediates. Nature 443, 713–716 (2006). 16964241 10.1038/nature05164

[b6] Daley J. M., Wilson T. E. & Ramotar D. Genetic interactions between HNT3/Aprataxin and RAD27/FEN1 suggest parallel pathways for 5′ end processing during base excision repair. DNA Repair (Amst) 9, 690–699 (2010). 20399152 10.1016/j.dnarep.2010.03.006 PMC3319107

[b7] El-Khamisy S. F. *et al.* Synergistic decrease of DNA single-strand break repair rates in mouse neural cells lacking both Tdp1 and aprataxin. DNA Repair (Amst) 8, 760–766 (2009). 19303373 10.1016/j.dnarep.2009.02.002 PMC2693503

[b8] Clements P. M. *et al.* The ataxia-oculomotor apraxia 1 gene product has a role distinct from ATM and interacts with the DNA strand break repair proteins XRCC1 and XRCC4. DNA Repair (Amst) 3, 1493–1502 (2004). 15380105 10.1016/j.dnarep.2004.06.017

[b9] Luo H. *et al.* A new XRCC1-containing complex and its role in cellular survival of methyl methanesulfonate treatment. Mol Cell Biol 24, 8356–8365 (2004). 15367657 10.1128/MCB.24.19.8356-8365.2004 PMC516742

[b10] Sykora P., Croteau D. L., Bohr V. A. & Wilson D. M. 3rd. Aprataxin localizes to mitochondria and preserves mitochondrial function. Proc Natl Acad Sci USA 108, 7437–7442 (2011). 21502511 10.1073/pnas.1100084108 PMC3088601

[b11] Bratic A. & Larsson N. G. The role of mitochondria in aging. J Clin Invest 123, 951–957 (2013). 23454757 10.1172/JCI64125 PMC3582127

[b12] Nunnari J. & Suomalainen A. Mitochondria: in sickness and in health. Cell 148, 1145–1159 (2012). 22424226 10.1016/j.cell.2012.02.035 PMC5381524

[b13] Schapira A. H. Mitochondrial diseases. Lancet 379, 1825–1834 (2012). 22482939 10.1016/S0140-6736(11)61305-6

[b14] Copeland W. C. & Longley M. J. Mitochondrial genome maintenance in health and disease. DNA Repair (Amst) 19, 190–198 (2014). 24780559 10.1016/j.dnarep.2014.03.010 PMC4075028

[b15] Fang E. F. *et al.* Defective mitophagy in XPA via PARP-1 hyperactivation and NAD(+)/SIRT1 reduction. Cell 157, 882–896 (2014). 24813611 10.1016/j.cell.2014.03.026 PMC4625837

[b16] Scheibye-Knudsen M. *et al.* Cockayne syndrome group B protein prevents the accumulation of damaged mitochondria by promoting mitochondrial autophagy. J Exp Med 209, 855–869 (2012). 22473955 10.1084/jem.20111721 PMC3328359

[b17] Sharma N. K. *et al.* Intrinsic mitochondrial DNA repair defects in Ataxia Telangiectasia. DNA Repair (Amst) 13, 22–31 (2014). 24342190 10.1016/j.dnarep.2013.11.002 PMC6211587

[b18] Valentin-Vega Y. A. *et al.* Mitochondrial dysfunction in ataxia-telangiectasia. Blood 119, 1490–1500 (2012). 22144182 10.1182/blood-2011-08-373639 PMC3286212

[b19] Scheibye-Knudsen M., Scheibye-Alsing K., Canugovi C., Croteau D. L. & Bohr V. A. A novel diagnostic tool reveals mitochondrial pathology in human diseases and aging. Aging (Albany NY) 5, 192–208 (2013). 23524341 10.18632/aging.100546 PMC3629291

[b20] Gueven N. *et al.* Aprataxin, a novel protein that protects against genotoxic stress. Hum Mol Genet 13, 1081–1093 (2004). 15044383 10.1093/hmg/ddh122

[b21] Akbari M., Visnes T., Krokan H. E. & Otterlei M. Mitochondrial base excision repair of uracil and AP sites takes place by single-nucleotide insertion and long-patch DNA synthesis. DNA Repair (Amst) 7, 605–616 (2008). 18295553 10.1016/j.dnarep.2008.01.002

[b22] Akbari M. *et al.* Overexpression of DNA ligase III in mitochondria protects cells against oxidative stress and improves mitochondrial DNA base excision repair. DNA Repair (Amst) 16, 44–53 (2014). 24674627 10.1016/j.dnarep.2014.01.015 PMC5156482

[b23] Maynard S., de Souza-Pinto N. C., Scheibye-Knudsen M. & Bohr V. A. Mitochondrial base excision repair assays. Methods 51, 416–425 (2010). 20188838 10.1016/j.ymeth.2010.02.020 PMC2916069

[b24] Frosina G., Cappelli E., Fortini P. & Dogliotti E. *In vitro* base excision repair assay using mammalian cell extracts. Methods Mol Biol 113, 301–315 (1999). 10443429 10.1385/1-59259-675-4:301

[b25] Santos J. H., Mandavilli B. S. & Van Houten B. Measuring oxidative mtDNA damage and repair using quantitative PCR. Methods Mol Biol 197, 159–176 (2002). 12013794 10.1385/1-59259-284-8:159

[b26] Castellotti B. *et al.* Ataxia with oculomotor apraxia type1 (AOA1): novel and recurrent aprataxin mutations, coenzyme Q10 analyses, and clinical findings in Italian patients. Neurogenetics 12, 193–201 (2011). 21465257 10.1007/s10048-011-0281-x

[b27] Liu P. *et al.* Removal of oxidative DNA damage via FEN1-dependent long-patch base excision repair in human cell mitochondria. Mol Cell Biol 28, 4975–4987 (2008). 18541666 10.1128/MCB.00457-08 PMC2519700

[b28] Szczesny B., Tann A. W., Longley M. J., Copeland W. C. & Mitra S. Long patch base excision repair in mammalian mitochondrial genomes. J Biol Chem 283, 26349–26356 (2008). 18635552 10.1074/jbc.M803491200 PMC2546560

[b29] Date H. *et al.* The FHA domain of aprataxin interacts with the C-terminal region of XRCC1. Biochem Biophys Res Commun 325, 1279–1285 (2004). 15555565 10.1016/j.bbrc.2004.10.162

[b30] Lakshmipathy U. & Campbell C. Mitochondrial DNA ligase III function is independent of Xrcc1. Nucleic Acids Res 28, 3880–3886 (2000). 11024166 10.1093/nar/28.20.3880 PMC110795

[b31] Reynolds J. J. *et al.* Defective DNA ligation during short-patch single-strand break repair in ataxia oculomotor apraxia 1. Mol Cell Biol 29, 1354–1362 (2009). 19103743 10.1128/MCB.01471-08 PMC2643831

[b32] Reynolds J. J., El-Khamisy S. F. & Caldecott K. W. Short-patch single-strand break repair in ataxia oculomotor apraxia-1. Biochem Soc Trans 37, 577–581 (2009). 19442253 10.1042/BST0370577

[b33] Podust L. M., Podust V. N., Sogo J. M. & Hubscher U. Mammalian DNA polymerase auxiliary proteins: analysis of replication factor C-catalyzed proliferating cell nuclear antigen loading onto circular double-stranded DNA. Mol Cell Biol 15, 3072–3081 (1995). 7760803 10.1128/mcb.15.6.3072 PMC230538

[b34] Sykora P. *et al.* Modulation of DNA base excision repair during neuronal differentiation. Neurobiol Aging 34, 1717–1727 (2013). 23375654 10.1016/j.neurobiolaging.2012.12.016 PMC5576894

[b35] Narciso L. *et al.* Terminally differentiated muscle cells are defective in base excision DNA repair and hypersensitive to oxygen injury. Proc Natl Acad Sci USA 104, 17010–17015 (2007). 17940040 10.1073/pnas.0701743104 PMC2040456

[b36] Williams J. S. & Kunkel T. A. Ribonucleotides in DNA: origins, repair and consequences. DNA Repair (Amst) 19, 27–37 (2014). 24794402 10.1016/j.dnarep.2014.03.029 PMC4065383

[b37] Reijns M. A. *et al.* Enzymatic removal of ribonucleotides from DNA is essential for mammalian genome integrity and development. Cell 149, 1008–1022 (2012). 22579044 10.1016/j.cell.2012.04.011 PMC3383994

[b38] Tumbale P., Williams J. S., Schellenberg M. J., Kunkel T. A. & Williams R. S. Aprataxin resolves adenylated RNA-DNA junctions to maintain genome integrity. Nature 506, 111–115 (2014). 24362567 10.1038/nature12824 PMC4064939

[b39] Kasiviswanathan R. & Copeland W. C. Ribonucleotide discrimination and reverse transcription by the human mitochondrial DNA polymerase. J Biol Chem 286, 31490–31500 (2011). 21778232 10.1074/jbc.M111.252460 PMC3173122

[b40] Murakami E. *et al.* Characterization of novel reverse transcriptase and other RNA-associated catalytic activities by human DNA polymerase gamma: importance in mitochondrial DNA replication. J Biol Chem 278, 36403–36409 (2003). 12857740 10.1074/jbc.M306236200

[b41] Yang M. Y. *et al.* Biased incorporation of ribonucleotides on the mitochondrial L-strand accounts for apparent strand-asymmetric DNA replication. Cell 111, 495–505 (2002). 12437923 10.1016/s0092-8674(02)01075-9

[b42] Cerritelli S. M. & Crouch R. J. Ribonuclease H: the enzymes in eukaryotes. FEBS J 276, 1494–1505 (2009). 19228196 10.1111/j.1742-4658.2009.06908.x PMC2746905

[b43] Caglayan M., Batra V. K., Sassa A., Prasad R. & Wilson S. H. Role of polymerase beta in complementing aprataxin deficiency during abasic-site base excision repair. Nat Struct Mol Biol 21, 497–499 (2014). 24777061 10.1038/nsmb.2818 PMC6168318

[b44] Akbari M. *et al.* Extracts of proliferating and non-proliferating human cells display different base excision pathways and repair fidelity. DNA Repair (Amst) 8, 834–843 (2009). 19442590 10.1016/j.dnarep.2009.04.002

[b45] Asagoshi K. *et al.* DNA polymerase beta-dependent long patch base excision repair in living cells. DNA Repair (Amst) 9, 109–119 (2010). 20006562 10.1016/j.dnarep.2009.11.002 PMC2819632

[b46] Prasad R., Dianov G. L., Bohr V. A. & Wilson S. H. FEN1 stimulation of DNA polymerase beta mediates an excision step in mammalian long patch base excision repair. J Biol Chem 275, 4460–4466 (2000). 10660619 10.1074/jbc.275.6.4460

[b47] Rass U., Ahel I. & West S. C. Actions of aprataxin in multiple DNA repair pathways. J Biol Chem 282, 9469–9474 (2007). 17276982 10.1074/jbc.M611489200

