# Supplementary material for: Slow mitochondrial repair of 5′-AMP renders mtDNA susceptible to damage in APTX deficient cells
Source: Sci Rep. 2015 Aug 10;5:12876. doi: 10.1038/srep12876 (PMC4530458; doi:10.1038/srep12876)
Supplement: Supplementary data [file srep12876-s1.pdf]

Slow mitochondrial repair of 5´-AMP renders mtDNA susceptible to damage in APTX deficient cells

Mansour Akbari[1], Peter Sykora[2], and Vilhelm A. Bohr[1,2,*]

[1]Center for Healthy Aging, SUND, University of Copenhagen, Denmark. [2]Laboratory of Molecular Gerontology, National Institute on Aging, 251 Bayview Blvd, Baltimore, USA.

* To whom correspondence should be addressed: Vilhelm A. Bohr, Laboratory of Molecular Gerontology, National Institute on Aging, NIH, 251 Bayview Blvd, Suite 100, Rm 06B133, Baltimore, MD 21224, USA. Tel.: 410 558 8162; fax: 410 558 8157; e-mail: vbohr@nih.gov

Supplementary Figure 1

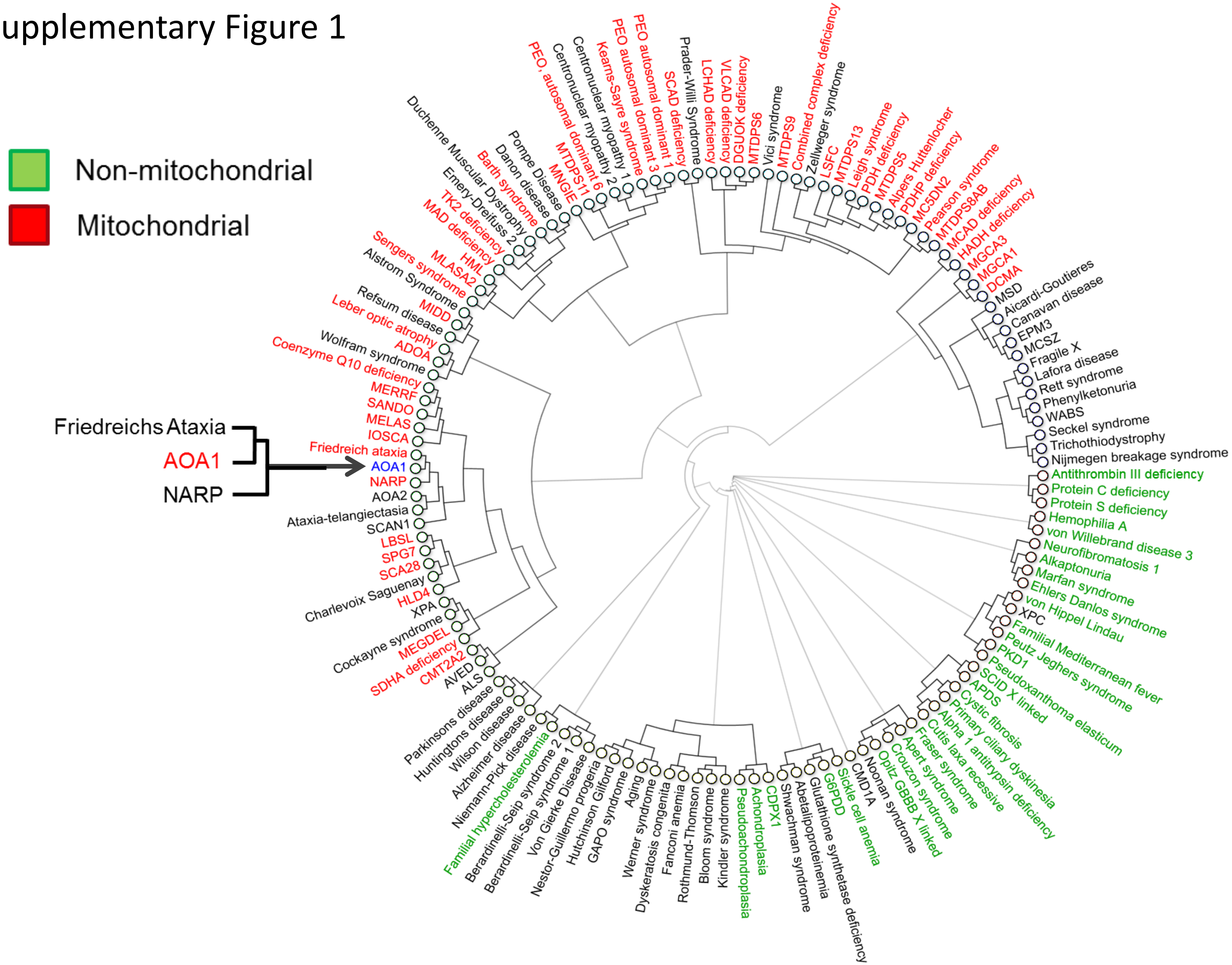

S1: AOA1 is more similar to mitochondrial diseases such as Friedreichs ataxia and NARP than to non mitochondrial diseases. Adapted from The mitochondrial disease database (www.mitodb.com). Refer to text for further information.

Supplementary Figure 2

(A)

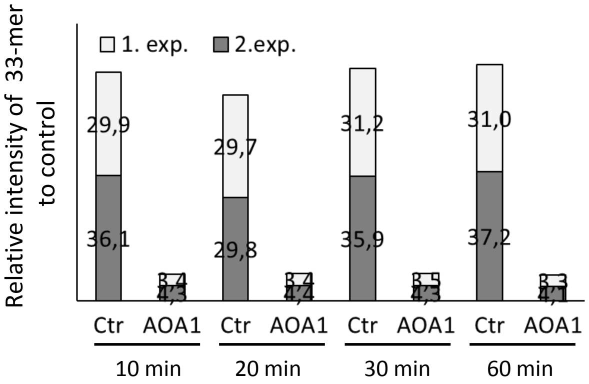

(B)

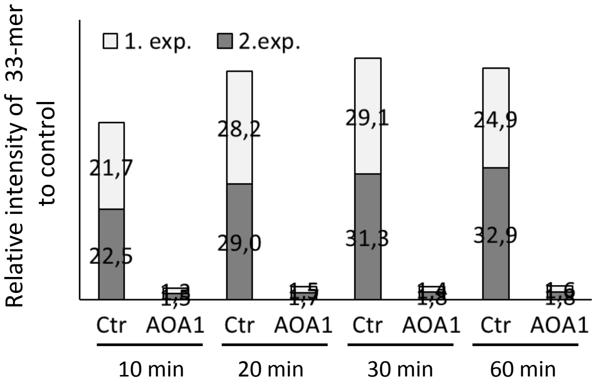

S2. Negatives (Figure 1C) correspond to the amount of DNA substrate used in each reaction and was used to calculate the rate of direct ligation of the nick in nuclear (A) and mitochondrial (B) extracts, respectively. The experiments were conducted in duplicate and the result of each experiment is shown in stacked column.

Supplementary Figure 3

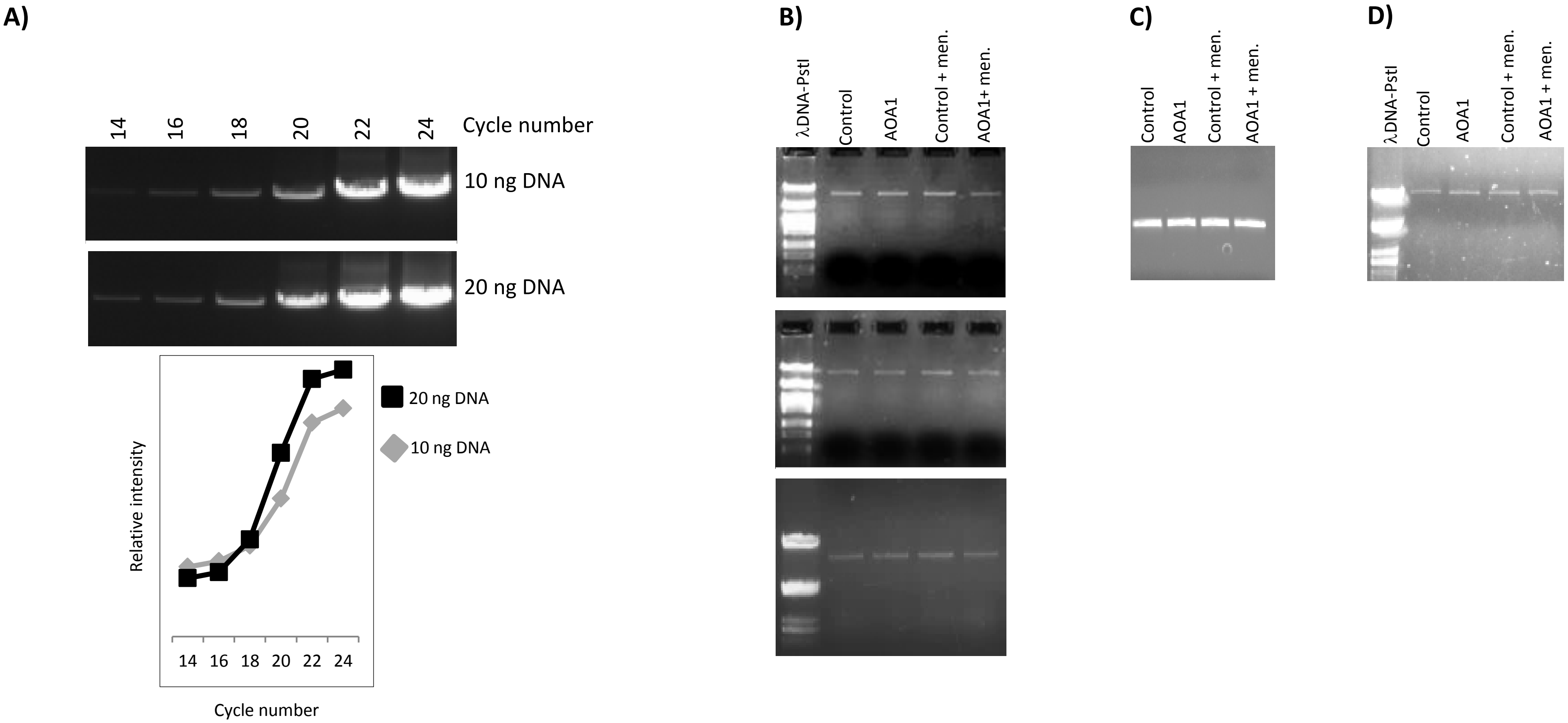

S3. PCR-based DNA damage analysis. (**A**) Experiment showing that at 20 cycle, PCR was within exponential amplification range. (**B**) PCR amplification of mtDNA (8.9 kb) in triplicate, (**C**) PCR amplification of a 179 bp fragment of mtDNA, and (**D**) PCR amplification of a 13.5 kb fragment of nuclear β-globin gene .
